# Nitrofurantoin-induced pulmonary fibrosis: a case report

**DOI:** 10.1186/1752-1947-2-169

**Published:** 2008-05-21

**Authors:** Natascha NT Goemaere, Karin Grijm, Peter ThW van Hal, Michael A den Bakker

**Affiliations:** 1 Departmentof Pathology, Josephine Nefkens Institute, Erasmus MC – University Medical Center Rotterdam, 3000 CA, Rotterdam, The Netherlands; 2 Department of Respiratory Medicine, Josephine Nefkens Institute, Erasmus MC – University Medical Center Rotterdam, 3000 CA, Rotterdam, The Netherlands; 3 Department of Respiratory Medicine, Spaarne Ziekenhuis, 2130 AT Hoofddorp, The Netherlands

## Abstract

**Introduction:**

Nitrofurantoin is a commonly used drug in the treatment and prevention of urinary tract infections. Many adverse effects of nitrofurantoin have been documented, including aplastic anemia, polyneuritis, and liver and pulmonary toxicity.

**Case presentation:**

We describe the clinical history and the autopsy findings in a 51-year-old woman with lung fibrosis of unknown etiology. She had a history of recurrent urinary tract infections, treated with nitrofurantoin for many years. She was referred to our hospital for screening for lung transplantation because of severe pulmonary restriction and dyspnea. Unfortunately, she died as a result of progressive respiratory insufficiency. At autopsy bilateral patchy, sharply circumscribed fibrotic areas in the upper and lower lobes of the lungs were seen with honeycombing. Microscopically, end-stage interstitial fibrosis with diffuse alveolar damage was observed. Due to the atypical distribution of the fibrosis involving both the lower and upper lobes of the lung, the microscopic pattern of the fibrosis and the history of long-term nitrofurantoin use, we concluded that this drug induced the lung fibrosis. The recurrent urinary tract infections were probably caused by a diverticulum of the urinary bladder, which was discovered at autopsy.

**Conclusion:**

This case shows that the use of nitrofurantoin may cause severe pulmonary disease. Patients with long-term use of nitrofurantoin should be monitored regularly for adverse pulmonary effects.

## Introduction

Drug-induced lung disease is a relatively common condition. Nitrofurantoin is one of the drugs known to be associated with adverse pulmonary reactions. Three types of reactions have been documented, acute, subacute and chronic, with various histological reaction patterns including pulmonary fibrosis. The acute form is more frequently reported in the literature than the chronic form. Here, we present the clinical history and autopsy findings of a 51-year-old woman with a history of lung fibrosis of unknown etiology. The fibrosis was atypical in its distribution and not readily compatible with typical forms of pulmonary fibrosis, and was finally attributed to the long-term use of nitrofurantoin.

## Case presentation

**Figure 1:**
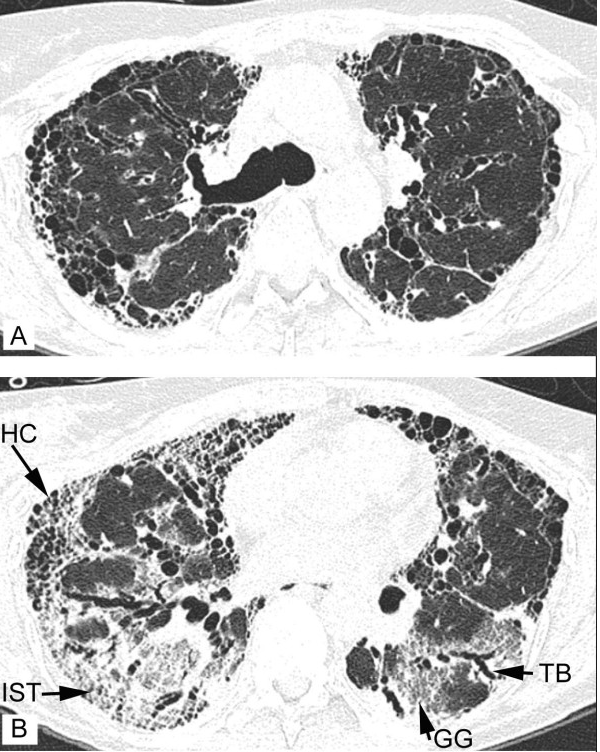
**High resolution computed tomography (HR-CT) of the lung showing characteristics of fibrosis**. (a) HR-CT at the level of the main carina. (b) HR-CT at the basal parts of the lungs 1 cm above the diaphragm. Inter- and intralobular septal thickening (IST), traction bronchiectasis (TB), honeycombing cysts (HC) and ground glass (GG) are indicated.

A 51-year-old woman was admitted with progressive shortness of breath. She had a 1-year history of rapidly progressive pulmonary restriction of unknown cause. A high resolution computed tomography (HRCT) scan revealed bilateral fibrotic changes, with traction bronchiectases, focal ground-glass opacities and honeycombing (Figures 1a and 1b). In addition, her medical history included polyneuropathy, fibromyalgia, hypercholesterolemia and recurrent urinary tract infections. The recurrent urinary tract infections had been treated with nitrofurantoin, prescribed at 15 mg daily for many years, resulting in a cumulative amount of over 140 g. Urological examination conducted 4 years prior to the current admission had not revealed any abnormalities of the bladder. Clinically, the differential diagnosis of the pulmonary fibrosis included an adverse reaction to the long-term use of nitrofurantoin, the result of an undiagnosed collagen-vascular disease or end-stage usual interstitial pneumonitis (UIP) (cryptogenic fibrosing alveolitis/idiopathic pulmonary fibrosis). Although the patient was taking many other drugs at the time, none of these has previously been shown to cause pulmonary fibrosis. Autoimmune serology, including assays for auto-antibodies against nuclear antigen, SS-A/SS-B, anti-cyclic citrullinated peptide (anti-CCP), anti-ds-DNA, Rnase protection (RNP), sphingomyelin (SM), antineutrophil cytoplasmic auto-antibodies (c-ANCA, p-ANCA), anti-Saccharomyces cerevisiae antibodies (ASCA IgA and ASCA IgG), was negative. Further blood chemistry analysis revealed no abnormalities; in particular there was no eosinophilia. Because of the severity of the pulmonary fibrosis, lung transplantation was considered. The screening examinations, performed 1 month prior to the current admission and including urological examinations, revealed no contraindications for transplantation. After the screening procedure the patient was discharged on glucocorticoids (prednisolone) and azathioprine. She was readmitted with sinusitis, onychomycosis and elevated liver enzymes [γ-glutamyl transferase (γGT) 363 U/l, aspartate-aminotransferase (ASAT; or glutamate-oxaloacetate-transaminase (GOT)) 45 U/l, alanine-aminotransferase (ALAT, or glutamate-pyruvate-transaminase (GPT)) 75 U/l and alkaline phosphatase (AP) 90 U/l]. On admission the chest X-ray (not shown) showed diffuse bilateral reticular nodular opacities. A liver biopsy showed mild hepatitis consistent with drug-induced hepatic injury. She died of respiratory failure after a febrile episode 16 days after admission. Permission for autopsy was obtained.

**Figure 2:**
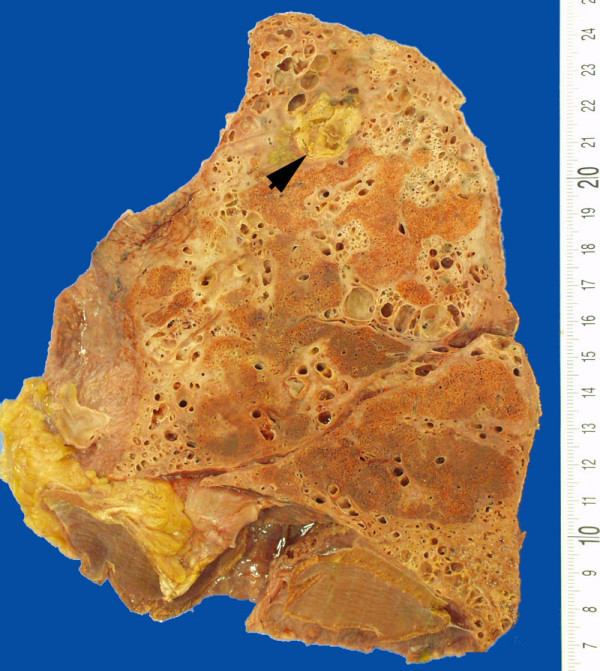
Transected lung showing randomly distributed areas of fibrosis with honeycombing and discrete islands of normal spongy lung tissue. Arrow: aspergilloma.

All clinical reports were collected and all previous histology was reviewed. A literature search for pulmonary fibrosis and nitrofurantoin therapy was performed. The post-mortem examination was performed according to the department's standard protocol. Samples from all organs were selected for histology, and the lungs in particular were sampled extensively. Routine hematoxylin and eosin slides were prepared from formalin-fixed, paraffin-embedded tissue blocks. Special stains for connective tissue (resorcin-fuchsin) and fungi (Grocott) were performed on selected blocks only. At post-mortem examination adhesions of the pleural membranes were noted both over the chest wall and diaphragmatic surfaces of the lung. There was no evidence of active pleuritis and there was no pleural effusion. Both lungs were firm with similar gross external appearances and with a similar aspect of the cut surface. Together the lungs weighed 1060 g. Macroscopically, irregular but sharply defined areas of residual spongy parenchyma, were surrounded by extensive areas of abnormal parenchyma. Here the lung tissue was firm and fibrotic with loss of spongy consistency and with extensive cystic changes, with cysts measuring up to 1 cm in diameter. The abnormal fibrotic parenchyma was evenly distributed over the upper and lower lobes (Figure 2). Parenthetically, the fibrotic areas were not distinctly located in sub-pleural or para-septal regions but were scattered throughout the lung lobes. There was no obvious peribronchial distribution of the inflammation. In the upper lobe of the right lung a sharply defined 1.5 cm cavity was observed filled with friable yellow tissue (Figure 2, arrow).

**Figure 3:**
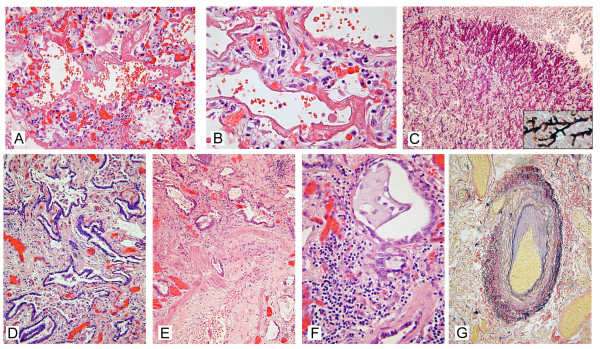
**Hematoxylin and eosin, periodic acid Schiff, Grocott and Elastica von Gieson stained sections of sampled lung tissue**. (a, b) Diffuse alveolar damage with denuded edematous alveolar septa with thick hyaline membranes and intra-alveolar hemorrhage. (c) Aspergilloma composed of radiating regularly branched fungal hyphae (periodic acid Schiff stain, insert: high magnification Grocott stain). (d, e, f) Lung parenchyma with severely distorted architecture consisting of established fibrosis with sharply angulated and cystically dilated bronchioli with inspissated secretions. In the interstitium a patchy lympho-histiocytic infiltrate is present (f). Bronchiolar smooth muscle hypertrophy (e) and squamous metaplasia (f) is seen.

Microscopically, there was a combination of diffuse alveolar damage (DAD) superimposed on end-stage interstitial fibrosis (Figure 3). In non-fibrotic lung tissue the alveolar septae were edematous with sloughing of pneumocytes. Hyaline membranes on denuded alveolar septae were readily identified. No granulomas were identified. Small intra-alveolar hemorrhages were present (Figures 3a and 3b).

The fibrosis appeared temporarily homogenous and mature, and composed of deeply eosinophilic staining collagen. Fibroblast foci were not seen. Within the fibrotic areas of the lungs extensive remodeling of the parenchyma was obvious, with cystic dilatation of bronchiolar structures and transformation of the alveolar architecture in which the residual alveoli were distended and lined by bronchial-type epithelium, consistent with honeycombing (Figures 3d and 3e). Squamous metaplasia was present in larger bronchioli. Within the cystically dilated air spaces inspissated secretions were present with an associated inflammatory histiocytic infiltrate admixed with neutrophils (Figure 3f). In the fibrotic interstitium the inflammatory infiltrate was composed of lymphocytes and histiocytes and patchy in its distribution. Reactive hypertrophy of bronchiolar smooth muscle was evident in areas of fibrosis; the inflammatory infiltrate frequently involved the walls of the bronchioles (Figure 3e). The pulmonary arteries showed mild to moderate medial hypertrophy and intimal thickening in the fibrotic parenchyma. There was no evidence of vasculitis. Outside the areas with fibrosis the vascular changes were minor (Figure 3g). The infiltrate was slightly more dense and diffuse in non-fibrotic lung tissue with features of diffuse alveolar damage. Eosinophils were not an important feature of the inflammatory infiltrate. In the right upper lobe an aspergilloma was seen (Figure 3c). In the pulmonary arteries fatty streaks were present, consistent with pulmonary hypertension.

The heart showed hypertrophy of both ventricles (right ventricular wall thickness 9 mm). The liver was congested and microscopic findings were similar to those seen in the biopsy. In the urinary bladder a large diverticulum of the posterior wall was observed with acute and chronic inflammation.

In summary, the post-mortem findings in this case show DAD superimposed on end-stage pulmonary fibrosis with signs of associated pulmonary hypertension and right-sided heart failure. In addition, an aspergilloma was present, which developed secondary to immunosuppressive therapy for pulmonary fibrosis. Finally, the DAD may well have been caused by urosepsis with its focus in the infected urinary bladder diverticulum. This diverticulum must have been the cause of the recurrent urinary tract infections for which long-term nitrofurantoin had been prescribed.

## Conclusion

The pattern of the interstitial disease did not fit with any of the typical entities. UIP was unlikely considering the absence of fibroblast foci, the even distribution over both the upper and lower lobes and the sparing of the para-septal and sub-pleural regions. Furthermore, the pattern and histology of the fibrosis did not meet the criteria of other recognized patterns of pulmonary fibrosis such as non-specific interstitial pneumonia or desquamative interstitial pneumonia. End-stage extrinsic allergic alveolitis was considered unlikely considering the absence of granulomas and the non-peribronchiolar distribution of the changes in the parenchyma. The serological investigations did not support lung fibrosis in the context of a collagen vascular disorder. Although histologically 'unclassified', the lung fibrosis seen in this case is considered an adverse effect of the long-term use of nitrofurantoin.

Diffuse interstitial (restrictive) lung diseases (DILDs) represent a heterogeneous group. They account for 15% of the non-infectious diseases seen by pulmonary physicians. Physiologically, DILDs are characterized by reduced oxygen-diffusing capacity, lung volume and compliance. In advanced DILD, histology is often non-specific and shows established fibrosis and honeycombing. The etiology of many of these restrictive diseases is not known. Identified causes of pulmonary fibrosis include environmental elements (asbestos, silica), radiation and drugs. Nitrofurantoin is one of those drugs; other examples are busulfan and bleomycin.

Nitrofurantoin is a broad-spectrum antibiotic used for clinical urinary tract infections, but may also be used in a prophylactic setting for patients with recurrent urinary tract infections. It is known to have several adverse effects such as aplastic anemia, polyneuritis, acute cholestatic and hepatocellular reactions, and pulmonary toxicity [1]. The acute form of nitrofurantoin toxicity is characterized by fever, cough and rapid onset of dyspnea [2]. The symptoms appear within 3 weeks of initiation of treatment. Chest X-rays show alveolar infiltrates. Symptoms often disappear rapidly after discontinuing nitrofurantoin treatment. Bronchoalveolar lavage (BAL) may be useful diagnostically, because an increased BAL fluid eosinophil percentage is found in 40% of the patients with interstitial lung disease and in 12% of the patients with drug-induced lung disease [3].

Chronic pulmonary nitrofurantoin toxicity is uncommon and may develop after 1 month to 6 years of nitrofurantoin treatment [1,2]. Chronic nitrofurantoin toxicity is more commonly seen in older patients and women and may spontaneously resolve after discontinuing antibiotic treatment [2].

Drug-induced lung diseases are relatively common and may result from various complex mechanisms. Different drugs may produce similar clinical syndromes and one drug may cause different types of reaction. Nitrofurantoin is a good example of a drug with many pulmonary manifestations including chronic or acute interstitial pneumonia, pulmonary hemorrhage, bronchoconstriction, anaphylaxis and pleural effusion [4]. The lung disease may be the result of direct toxicity or of indirect inflammatory and immunological processes. Direct toxicity in many cases is dose-related; a typical example of this is seen in bleomycin therapy. The toxic effect of a drug may be enhanced by several factors including decreased renal function and oxygen therapy. The pathological substrate begins with pulmonary edema, leading to DAD and eventually resulting in interstitial fibrosis. Radiographic diffuse lung opacities (reticular or reticulonodular) are seen, especially in the lung bases. HRCT scans reveal ground glass attenuation in combination with intralobular lines, traction bronchiectasis and honeycombing.

Another mechanism for pulmonary drug toxicity is a hypersensitivity reaction. This reaction is not dose-related and requires prior sensitization. The pathogenesis is an interaction between humoral antibodies or sensitized lymphocytes and the drug. Patients respond to withdrawal of the drug, but sometimes glucocorticoid therapy is needed. Other drug-induced pulmonary effects are pulmonary hemorrhage, bronchiolitis obliterans organizing pneumonia, lipoid pneumonia and pulmonary granulomas [4].

The most likely cause of pulmonary complications of nitrofurantoin therapy is a hypersensitivity reaction [5]. The interstitial pneumonitis induced by nitrofurantoin is now classified as a non-cytotoxic pneumonitis. Non-cytotoxic drugs, including nitrofurantoin, can activate lymphocytes. Those lymphocytes produce mediators that cause the release of many cytokines, resulting in a lymphocytic alveolitis. Another mechanism described in patients using nitrofurantoin is the disturbance of the equilibrium between oxidants and anti-oxidants in the lung. Nitrofurantoin induces an increased production of oxidants in the lung, resulting in the activation of several inflammatory responses [3]. In vitro experiments by Boyd et al. [6] revealed the production of toxic metabolic products of nitrofurantoin in the presence of oxygen and lung microsomes. The toxic products may cause lung injury and thus result in diffuse interstitial lung fibrosis [2]. This may also explain the prevalence of a chronic pulmonary reaction in the elderly. Many elderly people have a decreased creatinine clearance, which may result in accumulation of nitrofurantoin and its metabolites.

In conclusion, nitrofurantoin has its value in the treatment of urinary tract infections, but long-term use may be complicated by severe toxicity. Patients on long-term use of nitrofurantoin should be checked regularly for any complications and in particular for pulmonary fibrosis. Glucocorticoids may be beneficial in preventing fibrosis [2,3,5].

## Competing interests

The authors declare that they have no competing interests.

## List of abbreviations

BAL: bronchoalveolar lavage; DAD: diffuse alveolar damage; DILD: diffuse interstitial lung disease; HRCT: high resolution computer tomography; UIP: usual interstitial pneumonia.

## Consent

Written informed consent was obtained from the patient for publication of this case report and accompanying images. A copy of the written consent is available for review by the Editor-in-Chief of this journal.

## Authors' contributions

PTH and KG were the treating pulmonary physicians and prepared the manuscript. NG performed the post-mortem, undertook the literature survey and histological analysis and prepared the manuscript. MAB performed the post-mortem and histology and prepared the manuscript. All authors read and approved the final manuscript.
